# Experimental evidence for the enhanced and reduced stopping regimes for protons propagating through hot plasmas

**DOI:** 10.1038/s41598-018-32726-2

**Published:** 2018-10-01

**Authors:** S. N. Chen, S. Atzeni, T. Gangolf, M. Gauthier, D. P. Higginson, R. Hua, J. Kim, F. Mangia, C. McGuffey, J.-R. Marquès, R. Riquier, H. Pépin, R. Shepherd, O. Willi, F. N. Beg, C. Deutsch, J. Fuchs

**Affiliations:** 1 LULI–CNRS, CEA, École Polytechnique, Univ. Paris-Saclay, Sorbonne Univ., UPMC Univ. Paris 06, F-91128 Palaiseau cedex, France; 2 Institute of Applied Physics, 46 Ulyanov Street, 603950 Nizhny Novgorod, Russia; 3 Extreme Light Infrastructure - Nuclear Physics/Horia Hulubei National Institute for R&D in Physics and Nuclear Engineering, Bucharest-Magurele, 077125 Romania; 4 Dipartimento SBAI, Università di Roma “La Sapienza”, Roma, Italy; 5 Center for Energy Research, University of California, San Diego, La Jolla, CA 92093-0417 USA; 6 INRS-EMT, Varennes, Québec, Canada; 7 LPGP-Univ. Paris-Sud, (UMR-CNRS 8578), Orsay, France; 8 Lawrence Livermore National Laboratory, Livermore, CA 94550 USA; 9 High Energy Density Sciences Division, SLAC National Accelerator Laboratory, Menlo Park, CA 94025 USA; 10 ILPP, Heinrich-Heine Universität Düsseldorf, 40225 Düsseldorf, Germany

## Abstract

Our understanding of the dynamics of ion collisional energy loss in a plasma is still not complete, in part due to the difficulty and lack of high-quality experimental measurements. These measurements are crucial to benchmark existing models. Here, we show that such a measurement is possible using high-flux proton beams accelerated by high intensity short pulse lasers, where there is a high number of particles in a picosecond pulse, which is ideal for measurements in quickly expanding plasmas. By reducing the energy bandwidth of the protons using a passive selector, we have made proton stopping measurements in partially ionized Argon and fully ionized Hydrogen plasmas with electron temperatures of hundreds of eV and densities in the range 10^20^–10^21^ cm^−3^. In the first case, we have observed, consistently with previous reports, enhanced stopping of protons when compared to stopping power in non-ionized gas. In the second case, we have observed for the first time the regime of reduced stopping, which is theoretically predicted in such hot and fully ionized plasma. The versatility of these tunable short-pulse laser based ion sources, where the ion type and energy can be changed at will, could open up the possibility for a variety of ion stopping power measurements in plasmas so long as they are well characterized in terms of temperature and density. In turn, these measurements will allow tests of the validity of existing theoretical models.

## Introduction

The physics of fast-ion slowing-down in ionized matter (plasma) is a topic relevant to diverse areas of research and applications including solid-state physics^1–3^, astrophysics^2^, plasma strippers, plasma ion heating and fusion sciences^4,5^. However, a full and complete picture of this physics is still eluding us, due to both difficulties from the experimental and theoretical points of view.

Experimentally, there have been no more than a dozen reported measurements of ion stopping in plasmas^6–18^, all performed in different plasma conditions (density, temperature, composition) and involving different types of ions, from protons to much heavier (e.g. Ar, N) ions. The intrinsic difficulty in realizing such experiments is to couple a well-characterized ion beam to an equally well-known plasma with linear dimensions adequate to allow for a detectable energy loss. The ion beam is conventionally produced and characterized using accelerators^16^, ion diodes^10^ or ion-generated fusion reactions^14^. Characterizing the plasma, however, is much more difficult, especially for high-density plasmas where standard optical probes cannot penetrate^19^, and hence require X-ray^20–22^ probing, particle^21^ probing, or X-ray emission analysis^23^. A further difficulty is that a plasma, given its internal pressure, expands and cools on the nanosecond timescale^24^, which is also the typical duration of ion beams provided by accelerators^16^ and diodes^25^. This implies that usually the ion beam propagates through plasmas of varying temperature and density, an effect which must be deconvolved in order to retrieve the varying ion stopping power. To mitigate this issue, short-duration ion beams are necessary, which so far have only been possible using nuclear reactions-produced ion bursts (with typical 150–180 ps duration)^14^. However, only certain ions, with fixed energies, can be generated, thus preventing data collection over a wide range of projectile conditions.

Theoretically, calculating energy loss in a plasma of an ion with energy from a few keV up to hundreds of MeV (i.e. our regime of interest) requires evaluating the ion Coulomb interaction with both bound (if any) and free electrons, where the fractions of the two populations change depending on the density and temperature of the plasma. In a standard, simplified approach, typically used in hydrodynamic codes for inertial confinement fusion^24^, or more recently in a particle-in-cell code^26^, the stopping power due to bound and free electrons is treated independently and additively^27,28^. Standard treatments of free electron stopping, considering both close collisions^29^ and collective plasma effects are discussed in classical references^30^; however, there is a growing trend toward methods treating the two channels of energy transfer self-consistently^31–33^. Furthermore, while standard approaches are based on simplified models for Coulomb energy exchange, which require ad-hoc short and long distance cut-offs (resulting in the usual Coulomb logarithm), recent works aims at using a first principle description of the interaction^34^. And because the stopping powers calculated by the different procedures differ (see, e.g., Fig. 7 of ref.^5^, Sec. 3 of ref.^34^, Fig. 2 of ref.^15^) this points to the necessity of experimental data over a wide range of parameters to discriminate between different theoretical approaches.

Here we show that using short-pulse laser-accelerated ion beams^35,36^ is an attractive alternative to the experimental methods used to date. As of recently, laser produced ion beams routinely achieve particle energies of tens of MeV with more than 10^13^ particle in each bunch^35,36^. These ion beams are: (i) versatile in nature, since a simple change of target material exposed to the laser allows to change the species of the accelerated ions^37^, and (ii) have picosecond duration at the source^38^. These characteristics allows one to vary the ion type and to probe a pseudo-steady state plasma before the density and temperature conditions change appreciably. Since short-pulse laser-accelerated ion beams are usually broadband^35,36^, they experience debunching during flight, and hence temporal stretching while traveling from the source to the plasma target. Hence, the bandwidth of the probing ion beam needs to be decreased to keep the ion pulse duration short. In this work, we use a passive energy selector^39–41^ allowing the selection of the central energy and the bandwidth independently of the ion source. An advantage of this method is that the energy of the selected beam does not vary, even though the entire ion spectrum at the source may vary due to system variations such as shot-to-shot variability of the laser plasma interaction^42^. Here we achieved a proton beam duration as low as 75 ps, which ensured instantaneous probing of the plasma. Finally, using short-pulse laser-accelerated ion offers also the advantage of overall significant compactness compared to an accelerator.

A well-characterized dense target gas jet^43–45^ was heated from one side by a ns-duration high-power laser to produce a plasma. The high-density of the jet is necessary to slow down the probing protons sufficiently for detection. For our case, in partially ionized plasmas with a temperature of a few tens of eV, we observed, similarly as in previous studies, an enhanced stopping of the protons, when compared to stopping in non-ionized medium. However, for fully ionized and hot plasmas, we observed that the stopping in a plasma can be *reduced* to values lower than the stopping power in matter at ambient temperature, highlighting for the first time this stopping regime. Even though the plasmas used in the experiments are inhomogeneous and far from isothermal, the proton stopping measurements are found to be in agreement with simulations, using the laser-fusion hydrodynamic code DUED^46,47^, which includes plasma formation and heating. The simulations compute the collisional slowing down of protons through the simulated plasma to retrieve computer-simulated synthetic spectra of the protons, as recorded in the experiment. We should however emphasize that the agreement can be only viewed from a qualitative point of view since we were unable to fully constrain the plasma parameters (in density and temperature). This characterization would be required to quantitatively test the simulations and benchmark the used ion stopping model in the simulations.

## Considerations on proton (0.1 MeV-1 MeV) stopping power in a plasma with temperature up to a few hundred eV

Standard models (see^6,48^ for reviews) for *cold matter* predict the amount of energy transfer based on refined versions of Bohr’s pioneering and classical description of the interaction of the nucleus of the projectile with a target bound electron. The Bethe-Bloch formula^49^ predicts the stopping curve well in the energy range >0.1 MeV/u. At lower energies (not considered in this work), the Lindhard-Scharff-Schiott (LSS) model^50^ is commonly used. Theoretical and semi-empirical models for the stopping power are discussed at length in refs^6,48^, and all of these have been extensively tested for virtually all elements and a large number of compounds, using large databases of ion stopping powers in solids and gases at room temperature (see, e.g. the PSTAR^27^ and SRIM^48^ databases). In contrast, as mentioned earlier, there is only limited experimental data for ion stopping in plasmas.

To illustrate the differences between stopping in cold matter and in plasma, we will use the standard approach by adding the free- and bound-electron contributions, and using the simple model of Coulomb collisions. We refer here to only proton projectile energy in the range 0.2–1 MeV and plasma target temperature in the range 0–1000 eV. The main trends of the stopping power are uncovered by the following approximate expression for the stopping power of an elemental material, with atomic number *Z*, and average ionization degree Z*, for nonrelativistic protons^5,28^:

1 $${S}=-\,\frac{{dE}}{{\rho }\mathrm{dx}}=\frac{{K}}{{AE}}[({Z}-{{Z}}^{\ast }){{L}}_{{\rm{be}}}+{{Z}}^{\ast }{{L}}_{{\rm{fe}}}],$$

where the first and second terms in brackets refer to the contributions of bound electrons and free electrons, respectively. Here $K=\frac{2\pi {e}^{4}{N}_{A}{m}_{p}}{{m}_{e}}$, $E=\frac{{m}_{p}{v}_{p}^{2}}{2}$ is the projectile proton kinetic energy,*v*_*p*_ is the velocity, *A* is the target element mass number, *N*_*A*_ is the Avogadro number, and *L*_be_ and *L*_fe_ are the stopping numbers for electrons and ions, respectively (*e*: electron charge; *m*_*e*_: electron mass; *m*_*p*_: proton mass). For cold matter, with Z* = 0, Eq. (1) reduces to the Bethe-Bohr expression, which is known to reproduce experimental data fairly well for not-too-small proton energies (e.g., in Hydrogen for *E* > 0.1 MeV). In the limit of full ionization *Z* = Z*, we recover standard expression quoted in reference textbooks^29,30^. The leading terms of the relevant stopping numbers are given respectively by:

2 $${{L}}_{{\rm{be}}}=\,{ln}(\frac{2{{m}}_{e}{{v}}_{{\boldsymbol{p}}}^{2}}{\bar{{I}}})$$

3 $${{L}}_{{\rm{fe}}}={G}({x}){ln}(\frac{2{{m}}_{e}{{v}}_{{pe}}^{2}}{\hslash {{\omega }}_{{\rm{pe}}}})$$

where $\bar{{\rm{I}}}$ is the average atom (or ion) excitation energy, ${\omega }_{{\rm{pe}}}={(4\pi {n}_{e}{e}^{2}/{m}_{e})}^{1/2}$ the electron plasma frequency, *n*_*e*_ the electron number density, *v*_pe_ the average relative speed between fast proton and plasma electrons, *e* the unit charge,

4 $${x}=\frac{{{v}}_{{p}}}{\sqrt{\frac{2{k}{{T}}_{{e}}}{{{m}}_{{e}}}}}=\sqrt{\frac{{{m}}_{{e}}}{{{m}}_{{p}}}}\sqrt{\frac{E}{k{T}_{e}}}=23.3\,\sqrt{\frac{{E}({MeV})}{{{T}}_{{e}}({eV})}}$$

is the ratio of the fast proton velocity to the electron thermal velocity and the function *G* is given by:

5 $${G}({x})={\rm{erf}}({x})-\frac{2}{\sqrt{{\pi }}}{x}\,\exp (\,-\,{{x}}^{2}).$$

Asymptotically, *G*(*x*) ≈ 1 for $x\gg 1$, and $G(x)\approx \frac{3{x}^{3}}{4}$ for *x* ≪ 1. The average excitation energies, $\bar{{\rm{I}}}$, are obtained from data tables^6^ for neutral atoms. In particular, <I> = 19.2 eV for Hydrogen and $\bar{{\rm{I}}}$ ≈ 190 eV for Argon. Average excitation energies of partially stripped ions are higher than for atoms since such ions have already lost the outer shell electrons^5,28^. Eqs (2) and (3) refer to the quantum limit of the minimum impact parameter (maximum momentum exchange) appearing in the Coulomb logarithm, which is appropriate when $\frac{{e}^{2}}{\hslash {v}_{p}}\ll 1$^30^, i.e. for proton energy *E* ≫ 25 keV. At the densities of interest (*n*_*e*_ ≤ 10^21^ cm^−3^), the plasmon energy $\hslash {\omega }_{{\rm{pe}}}=3.71\times {10}^{-11}\sqrt{{n}_{e}(c{m}^{-3})}{\rm{eV}}$ is much smaller than the average excitation energy, $\bar{{\rm{I}}}$. Also, for plasma temperatures up to a few tens of eV and proton projectile energies of a few hundred keV, $x\gg 1$ (see Eq. 4), hence *G*(*x*) ≈ 1, and then ${L}_{{\rm{fe}}}\gg {L}_{{\rm{be}}}$, i.e. the free electron stopping number is significantly larger than the bound electron stopping number.

**Figure 1:**
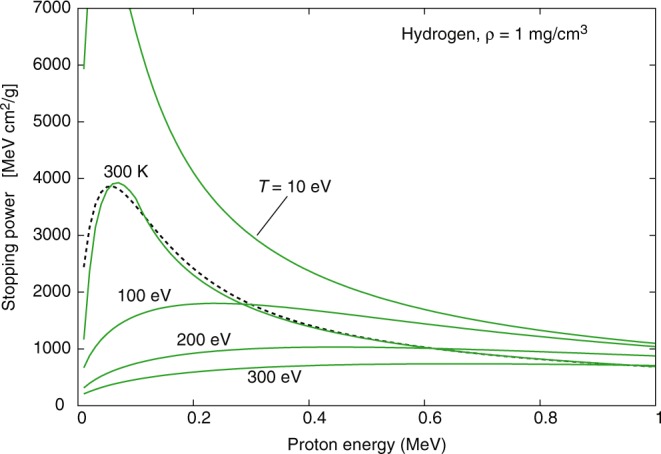
Calculated stopping power for protons of energy E < 1 MeV in Hydrogen at density of 1 mg/cm^3^ and different temperatures (solid curves). The dashed curve refers to SRIM data^48^ for gaseous Hydrogen at room temperature.

**Figure 2:**
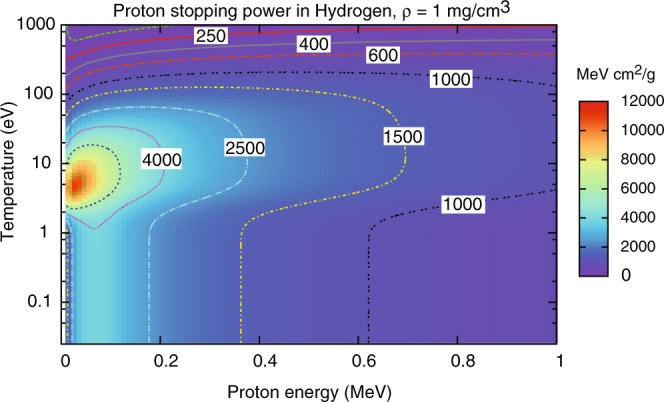
Calculated stopping power for protons in Hydrogen at density of 1 mg/cm^3^: color map and iso-stopping power contours in the proton energy - Hydrogen temperature plane.

**Figure 3:**
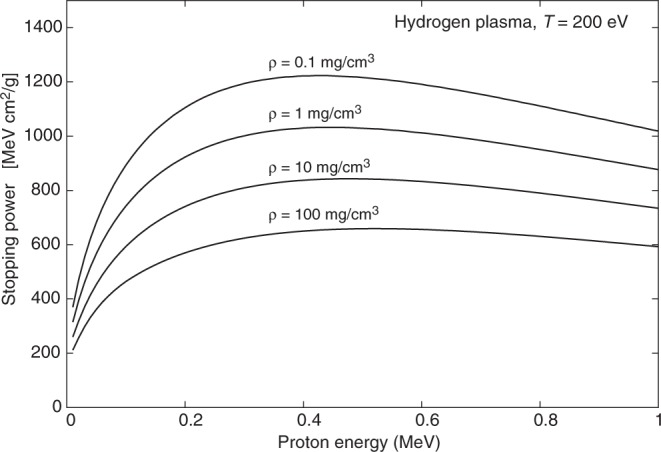
Calculated stopping power for protons of energy E < 1 MeV in Hydrogen plasma at 200 eV, and different densities.

**Figure 4:**
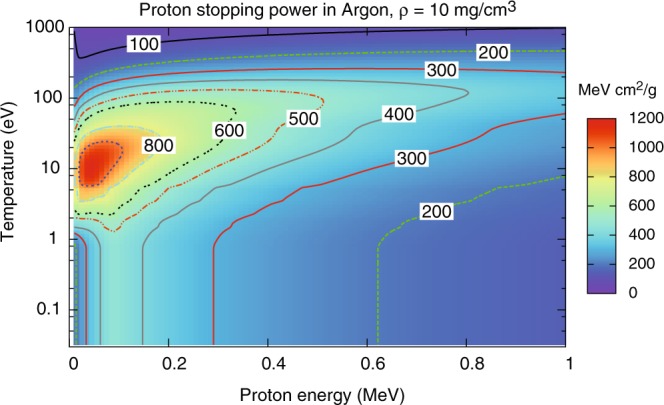
Calculated stopping power for protons in Argon at density of 10 mg/cm^3^: color map and iso-stopping power contours in the proton energy - Argon temperature plane.

It follows that as soon as ionization occurs (typically at temperatures of the order of 1 eV for Hydrogen) the stopping power increases substantially with respect to the cold matter value. At higher temperatures, however, the average velocity of the plasma electrons becomes comparable to or exceeds that of projectile protons, hence *x* < 1 (see Eq. (4)), and the stopping power decreases again; these temperature effects are clearly illustrated in Fig. 1 and Fig. 2. The free electron density also affects plasma stopping power, as ${\omega }_{{\rm{pe}}}\propto \sqrt{{n}_{e}}$ and then *L*_fe_ (and hence the stopping power) decreases with increasing density, as clearly shown in Fig. 3. Figures 1–3 refer to Hydrogen; similar temperature effects are found for Argon, as shown by the stopping power map of Fig. 4. Figures 1–4 have been obtained using the stopping power model included in the code DUED. This model adds the contributions of cold matter and plasma using standard expressions for the stopping numbers; for the dependence of the average excitation energy on ion charge state, the heuristic expression suggested by Melhorn^5^ is used. Ionization is computed by an average atom model. For all elements but Hydrogen, at low proton energy, the cold matter stopping power is taken as the minimum between Bethe’s and Lindhard’s values (but this does not affect the simulations presented in this paper). For the cold stopping in Hydrogen (again, without any effect on the present simulations) and proton energy *E* < 0.1 MeV, DUED uses a smooth fit to experimental data.

## Results

### Setup of the experiments

The results were obtained during two experiments that were performed using the ELFIE laser at the Laboratoire pour l’Utilisation des Laser Intenses (LULI) and the TITAN laser at the Jupiter Laser Facility at the Lawrence Livermore National Laboratory. The experimental platform was the same; however, we note that the technique used to accelerate the probe proton beam differed between the two experiments, but did not impact the result of our investigation since the energy selector was implemented in between proton the source and the plasma medium. Furthermore, in the interest to produce hot plasmas, the laser energy available at TITAN for heating the plasma was higher.

**Figure 5:**
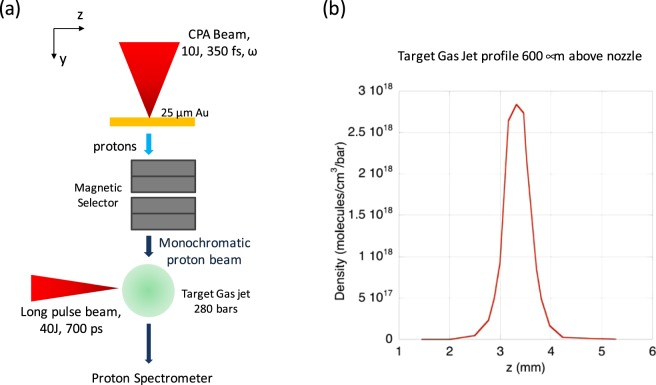
(**a**) Top view of the experimental setup at the ELFIE laser, (**b**) The target jet profile 600 μm above the nozzle, which corresponds to the location to which the proton beam passed in the target jet.

The general experimental setup is shown in Fig. 5. In the experiment performed at the ELFIE laser, the proton beam was generated through the Target Normal Sheath Acceleration (TNSA) mechanism^51^ by a short pulse beam with 10 J, wavelength of 1.058 μm, 350 fs pulse length, irradiating a 10 µm thick Au foil with an intensity greater than 10^19^ W/cm^2^. The main diagnostic was a proton spectrometer which consisted of an entrance slit that, limited the viewing angle of ~0.01 str and a pair of permanent magnets with a B-field of ~0.5 Tesla. The detector used was a FujiFilm TR image plate; it was scanned using a FujiFilm FLA-7000 scanner. The calibration curve published in Mori *et al*.^52^ and Mancic *et al*.^53^ were then used to convert the raw values from the scanner to proton number.

The proton beam generated had a broadband energy spectrum with a cutoff energy around 10 MeV. At the TITAN laser, the proton beam was created by irradiating a Hydrogen gas jet with 150 J, 700 fs laser and accelerating the particle beam orthogonal to the laser axis by Coulomb explosion^54^. Here, the proton beam had a broadband spectrum up to several MeV. In both cases, the emitted proton beams were sent through an energy selector device outputting a spectrally-narrow beam on the same axis as the injected proton beam^39,40^. The selector was set such that the outgoing proton beam had a bandwidth of *ΔE/E* = 12.5% at ELFIE and 2% at TITAN. The number of ions in the bunch (>10^9^ particles) is high enough to allow for energy loss measurements, but at the same time low enough to not perturb the heated plasma.

The fast protons then crossed a dense target plasma jet that was produced by a supersonic nozzle^55^ with a 1 mm diameter exit hole, to produce a Gaussian density profile, attached to a Clark-Cooper gas valve and a Haskel booster compressor. Two types of gases as targets were tested: Hydrogen and Argon. The backing pressure was 280 bars. We note that, in the case of the Argon target, it is likely to be aggregated into nanometer-size clusters upon exit of the nozzle. However, this should not affect stopping power measurements. Indeed, cold material energy loss essentially depends on the average density of the target material, and clusterization might at most slightly affect the average excitation potentials. Laser-irradiated jets are heated at such temperatures that no clusterization may still occur.

The target gas jet was used in its neutral form, in which case the measurements were found in reasonable agreement with existing database of stopping power in cold medium, as already reported in ref.^43^. Following this, the target gas jet was heated and ionized by a second, ns-duration laser pulse (see Fig. 5). At ELFIE, this laser was of 40 J, 700 ps FWHM duration; at TITAN, it was up to about 300 J in a pulse of 2 ns (1 ns plateau surrounded by two ramps of 0.5 ns).

**Figure 6:**
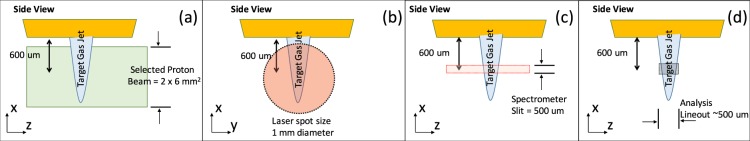
Side view of the experimental setup at ELIFIE to illustrate the sizes and shapes of the interaction. (**a**) The proton beam transverse profile. (**b**) The heating laser spot size. (**c**) The proton spectrometer slit size and position after the target jet. (**d**) The lineout off of the spectrum as recorded by the detector.

More precise views of the experimental parameters are shown in Fig. 6 for the experiment performed at ELFIE. The proton beam with a transverse profile of 2 × 6 mm^2^ was centered at 600 μm below the nozzle of the target jet, as shown in Fig. 6a; this was verified using an image plate after the beam with a thin light-tight filter^39^. The target gas jet density profile in the plane normal to the jet axis had been measured, by optical interferometry and using lower backing pressure, to have a Gaussian shape, with a FWHM of 650 μm and on-axis density n_0_ = 6 × 10^20^ atoms/cm^3^, in the plane 600 μm below the nozzle. The on-axis jet density was found to decrease roughly linearly with the distance from the nozzle, for distances between 300 and 900 μm, with (Δ*n*/*n*_0_)/*L* ≈ (20%)/600 μm.

The proton bunch passed through the target jet with a time-of-flight (TOF) bunch length of ~500 ps. The heating laser had a spot size of 1 mm diameter, centered 600 μm below the nozzle, as shown in Fig. 6b. A proton spectrometer was used to measure the transmitted proton beam energy, i.e. it was pointed 600 μm below the nozzle and had a horizontal slit width of 500 μm, as shown in Fig. 6c. In Fig. 6d, we show the spectrum selected for analysis was centered on where the proton beam had passed, i.e. through the center of the target jet. Thus, the analysis lineout width was 500 μm at the target jet plane, i.e. compensated for the magnification effect inside the spectrometer.

**Figure 7:**
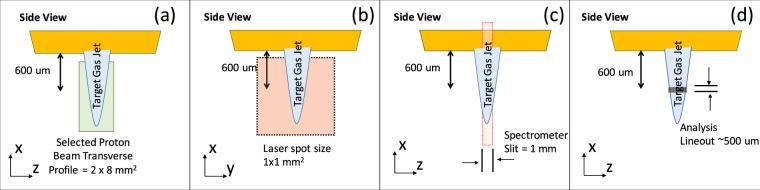
Side view of the experimental setup at TITAN to illustrate the sizes and shapes of the interaction. (**a**) The proton beam transverse profile. (**b**) The heating laser spot size. (**c**) The proton spectrometer slit size and position after the target jet. (**d**) The lineout off of the spectrum as recorded by the detector.

The experimental parameters for the TITAN experiment are shown in Fig. 7. The proton beam with a transverse profile of 2 × 8 mm^2^ was centered at 600 μm below the nozzle of the target jet, as shown in Fig. 7a; this was verified again using an image plate after the beam with a thin light-tight filter. The proton bunch that passed through the target jet had a TOF bunch length of ~75 ps. The heating laser had a spot size of 1 × 1 mm^2^ square, also centered 600 μm below the nozzle, as shown in Fig. 7b. The proton spectrometer to measure the altered proton beam energy was pointed 600 μm below the nozzle and had a slit width of 1 mm, as shown in Fig. 7c. The spectrum selected for analysis was centered on where the proton beam had passed through the center of the target jet and the analysis lineout width was 500 μm at the target jet plane, as shown in Fig. 7c, i.e. compensated for the magnification effect inside the spectrometer. Also, for this experiment, an X-ray spectrometer^56^, utilizing a flat field grating with a bandwidth of 200–2000 eV, was setup to look at Argon L-shell lines to determine the plasma temperature of the heated target plasma jet. There is a slit in front of the spectrometer that provided spatial resolution of about 1 mm at the source and since the detector was also a FujiFilm TR image plate, the recorded spectra is time-integrated.

### Measurement of enhanced stopping in partially ionized Argon

**Figure 8:**
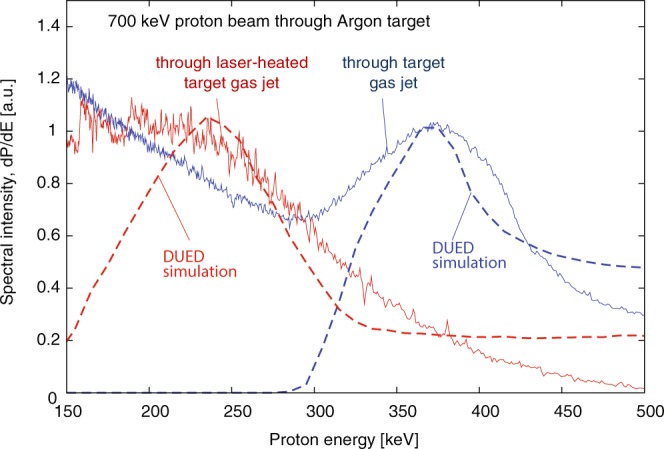
Experimental (solid) and simulated (dashed) energy spectra of a proton beam with energy of 700 keV, passing through a neutral and cold Argon target gas jet (blue) and a laser-heated (at TITAN) Argon target plasma jet (see main text for laser and jet parameters).

The measurement of the interaction of protons with energy of 700 keV with an Argon target gas jet, performed at the TITAN facility, is shown in Fig. 8. Here the spectra are presented with normalized units for ease of comparison; normalization was done at the very end, i.e. after conversion into proton number and after background subtraction. Argon was selected such that the gas was only partially ionized with the employed laser heating parameters, in order to be able to observe the effects of the so-called “enhanced stopping”, as was already highlighted in all previous experimental investigations of ion stopping in plasmas. In the shot we discuss here, the Argon jet was heated by a TITAN laser pulse of 315 J. The output proton spectra from the neutral target gas jet and the laser-heated target plasma jet are both shown in Fig. 8 (the input spectrum is not shown to focus on the downshifted spectra, but is similar to the input spectrum shown in Fig. 10, and even spectrally narrower). The energy change in the proton beam is consistent with what is predicted by the values in PSTAR as it gives a ΔE = 310 keV. With a plasma target, the proton beam suffers significantly larger energy loss in the plasma than in the cold target gas. It should be noted that the signal of the measured spectrum presented in Fig. 8 has an artificially high background at energies less than 200 keV. This is due to the calibration function used to convert the measured raw signal to proton number that amplifies noise as the proton energy lowers^52^. We verified that performing this conversion in units did not however change the location of the peak of the spectrum above 200 keV.

The recorded spectrum from the X-ray spectrometer was analysed with the collisional radiative atomic code FLYCHK^57^ and showed that the Argon plasma electron temperature ranged from 150–200 eV with an electron density of 10^18^–10^19^ 1/cm^3^. The purpose of this spectrometer is not to give exact plasma characteristics as there are gradients and opacity issues, but provides an order of magnitude to guide the analysis, detailed below, performed with the hydrodynamic code.

**Figure 9:**
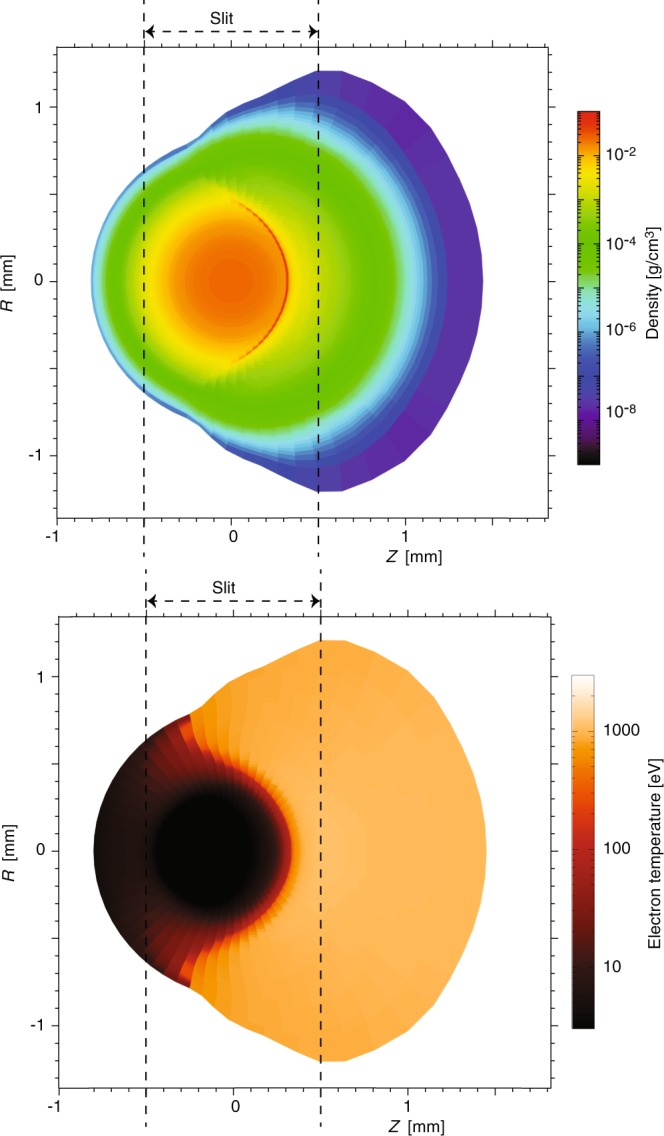
For the same experiment as in Fig. 8, density and temperature maps at the time of the interaction with the proton beam, as simulated using the DUED code (see text for details). The figure also shows the positions of the slit limiting the collected proton beam (as illustrated in Fig. 7c).

Synthetic proton spectra were produced by computing proton energy loss through the laser heated target plasma using the two-dimensional Lagrangian radiation-hydrodynamics code DUED^46^. To simulate the laser heating, the code employs a two-temperature hydrodynamics model and multi-group radiation diffusion. The laser-plasma interaction is included via ray-tracing. Plasma refraction and inverse Bremsstrahlung absorption are taken into account. Simplifications have been introduced to model the considered intrinsically three-dimensional experiments, in which a target gas jet is irradiated by a nearly cylindrical laser pulse orthogonal to the jet axis and crossed by a proton beam orthogonal to both the jet axis and the laser axis. The simulations were performed in a plane orthogonal to the jet axis, assuming cylindrical symmetry around the laser beam. The simulated target was therefore initially spherical, with Gaussian density profiles. With reference to Fig. 9, the laser propagates along the *Z*-axis (horizontal in the picture), while protons move along constant-*Z* lines (vertical in the Figure).

The code DUED was then also used to trace projectile protons through the plasma at t = 900 ps. It is assumed that protons (with an assigned initial spectrum) propagate normal to both laser and jet axis. The synthetic spectra integrate, as in the experiment, the whole proton beam crossing the target plasma jet over the slit width (as is illustrated in Fig. 7c). The width of the slit is indicated by dashed line in Fig. 9. The synthetic spectra are also integrated along the same vertical slice of the target plasma jet as in the experiment (as is illustrated in Fig. 7d). To account for the variation of the jet density with the distance from the nozzle, simulations are performed for different jet densities and the synthetic spectrum of protons is obtained by appropriately adding and weighting the spectra from the different simulations.

The simulation parameters are as follows: Gaussian radial density profile, with FWHM = 600 μm, peak mass density of 3.4 × 10^−2^ g/cm^3^ (5 × 10^20^ atoms/cm^3^), laser pulse of 2 ns, with a central plateau of 1 ns preceded and followed by 0.5 ns ramps, peak intensity of 2.3 × 10^13^ W/cm^2^, spot diameter of 1 mm, and total energy of 310 J. Simulated density and temperature maps at t = 1 ns are shown in Fig. 9. We see that only a fraction of protons crosses the 500–800 eV hot plasma corona. Most protons instead cross a layer of low-to-moderate temperature, compressed plasma. Due to the high atomic number, and hence the higher temperatures required for complete ionization, even at temperatures of 200–300 eV the stopping power of such a plasma is higher than that of cold matter for 500 keV protons (see Fig. 4). As a result, most of the protons suffer larger energy losses than in the cold gas and the detected spectrum moves towards lower energies; this effect can be well seen in the experimental and simulated spectra shown in Fig. 8.

### Measurement of reduced stopping in hot Hydrogen

**Figure 10:**
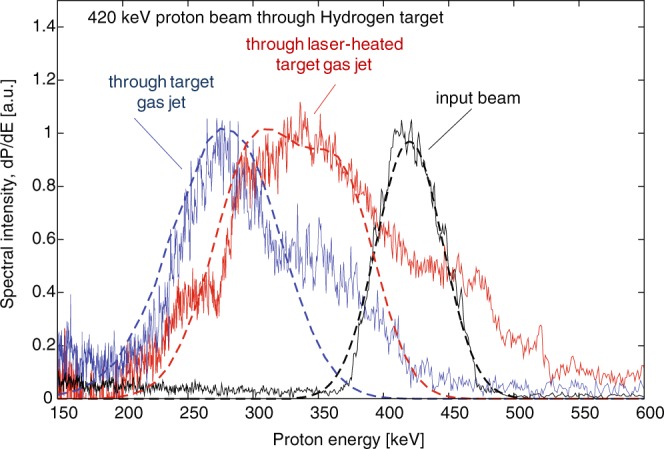
Experimental (solid) and simulated (dashed) energy spectra of a proton beam with energy of 420 keV, passing through a Hydrogen target gas jet (blue) and a laser-heated (at ELFIE) Hydrogen target plasma jet (see main text for laser and jet parameters). The input proton spectrum is also shown (black).

The measurement of the interaction of protons with energy of 420 keV with a Hydrogen jet, performed at the ELFIE facility, is shown in Fig. 10. The spectra of the input beam and of the output beam, in the unheated and heated target gas jet, are shown in Fig. 10. In the unheated case, the experimental spectrum peaks at about 286 keV, indicating an energy loss of 234 keV for the 420 keV protons crossing the jet along a diameter. This result is consistent with predictions of PSTAR^27^ database. The output proton spectrum of the heated case is shown by the red curve of Fig. 10. It should be noted that the low energy portion of the spectrum in Fig. 10 does not have an artificially high background as in Fig. 8. This is due to the fact that the signal-to-noise ratio was in this case much higher, thus the application of the calibration function to convert the raw data to proton number did not strongly affect the spectral shape at low energy. In any case, it is apparent that the energy loss is smaller than in the cold gas case.

**Figure 11:**
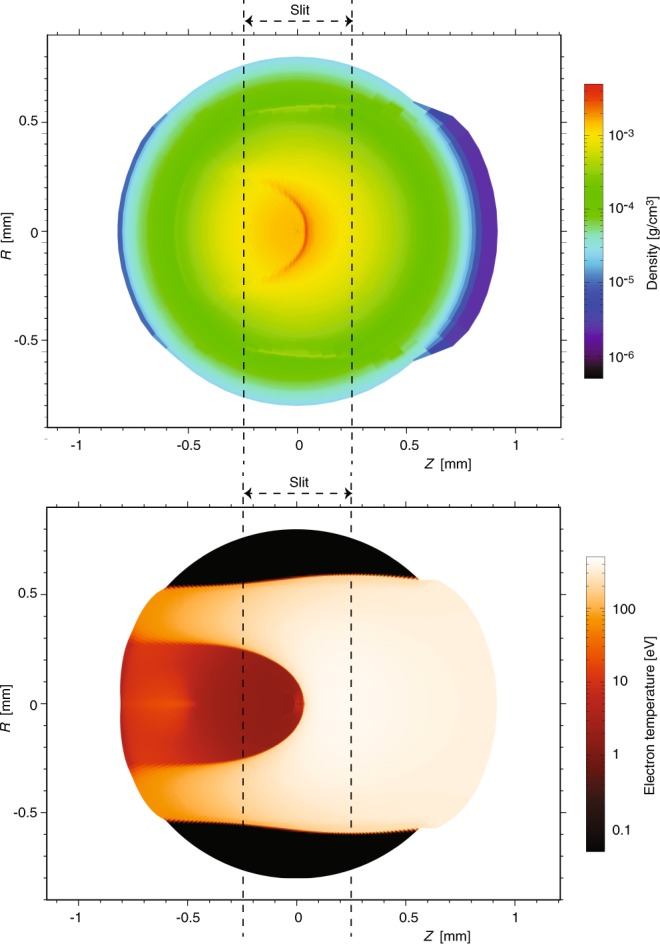
For the same experiment as in Fig. 10, density and temperature maps at the time of the interaction with the proton beam. The figure also shows the position of the slit limiting the collected proton beam.

Again, as for the enhanced stopping case in Argon, DUED was here used to simulate the heating of the Hydrogen target gas jet. For the experiment performed at ELFIE and the Hydrogen target gas target, the inputs to the simulation are the following: Gaussian radial density profile, with FWHM = 600 μm, peak mass density of 1.5 × 10^−3^ g/cm^3^ (4.5 × 10^20^ molecules/cm^3^), laser peak intensity of 1.1 × 10^13^ W/cm^2^, Gaussian temporal profile with FWHM of 600 ps and total energy of 42J). The simulation results at t = 800 ps, i.e. around the time of proton beam interaction, is shown in Fig. 11. The results show that in our experiments the produced plasma is highly inhomogeneous, as expected, and even with a small shocked region, however, the laser heated region is approximately isothermal (with temperature between 150–200 eV) and shows “gentle” density variations.

The simulated spectra of the proton beam passing through the cold gas jet and the laser-heated plasma calculated by DUED, are shown in Fig. 10. Note that, when comparing the simulated spectra to the experimental ones, we observe in the latter signal in other parts of the spectra, namely near the energy of the input beam and energies between the input beam energy and the strongest part of the spectrum at lower energy. We believe that this is likely caused by the input beam partially not crossing the highest density part of the gas/plasma column and scattering. Although many features of the experimental spectra cannot be reproduced, it is found that the synthetic spectra and the experimental spectra qualitatively agree in the region of peak intensity, i.e. displaying a clear, as in the experiment, reduced stopping behavior compared to the propagation through the neutral gas.

## Conclusions and Perspectives

We have demonstrated, using experiments and simulations, the feasibility of performing proton stopping measurements in a plasma target using short-pulse laser accelerated protons and a passive energy selector to reduce the energy bandwidth of the proton bunch. It allows the study of the slowing down of protons with energy of 400–800 keV in hot, fully ionized Hydrogen and in partially ionized Argon plasmas on a sub-nanosecond time scale. The measurements herein, however, does not allow us to directly benchmark proton stopping codes since the analysis still relies heavily on the plasma evolution predicted by hydrodynamic codes.

This technique, which is by far more compact than using conventional ion accelerators, or ions produced by nuclear reactions, has some further advantages: (i) the nature of the ion, the stopping of which is desired to be measured, can be changed by changing the target exposed to the laser, (ii) the energy of the ion can be easily tuned by adjusting the passive energy selector, and (iii) the duration of the ion pulse injected in the plasma can be set to be much shorter than the plasma hydrodynamic time-scale, allowing to probe it in a snapshot. We have shown that with such a technique we could observe the enhanced stopping regime in partially ionized plasmas, as revealed in previous experiments, but we have also shown for the first time the reduced stopping regime predicted by theory for hot plasmas.

The measurements we reported here were limited in the number of shots we could perform, mainly due to the allocated beam-time on the ELFIE and TITAN laser facilities. Hence the ideal scan of various proton energies, probing ion type and plasma parameters, was not possible. The major improvement that upcoming large-scale laser facilities will experience in terms of repetition rate^58^ combined with recently demonstrated high repetition rate proton sources^59–61^ will greatly improve the statistics as required for precision measurements. The measurements are also limited, for the same reasons, in the characterization of the plasma we could perform. Characterizing *in situ* the density is not possible employing usual visible light probes, due to the refraction in the gradients of the high-density target plasma jet we used^19^; for this we would need to use X-ray probes, hence additional laser beam and diagnostic setup. Finally, to improve in precision and control the gradients of the target plasma, one way would be to resort to particle^62^ or X-ray heating, instead of laser, of the target. Due to their deep penetration, they allow heating solid-density or even compressed materials in a fast and controlled manner. For X-rays, XFEL facilities, with their high photon number in extremely short pulses, allow now to do so and reach high temperatures in plasmas^63–65^.
